# Perceptions and experiences of maternity care workers during COVID-19 pandemic in Lagos State, Nigeria; a qualitative study

**DOI:** 10.1186/s12913-022-08009-y

**Published:** 2022-05-06

**Authors:** Charlotte Leung, Tolulope Olufunlayo, Zahra Olateju, Christine MacArthur, Beck Taylor

**Affiliations:** 1 College of Medical and Dental Sciences, University of Birmingham, Birmingham, UK; 2 Department of Community Health & Primary Care, College of Medicine, University of Lagos, Idi-Araba, Lagos, Nigeria; 3 Institute of Applied Health Research, Public Health, Epidemiology and Biostatistics, School of Health and Population Sciences, University of Birmingham, Birmingham, UK

**Keywords:** Covid-19, Nigeria, Maternity care, Qualitative, Midwives, Traditional birth attendants, Perceptions, Experiences

## Abstract

**Background:**

The COVID-19 (coronavirus disease 2019) pandemic challenges provision and access to essential maternity care in low-resilience health systems. The aim of this study was to explore maternity healthcare workers’ experiences of, and perceptions about providing maternity care during the COVID-19 outbreak in Lagos State, Nigeria.

**Methods:**

This qualitative study conducted individual, remote, semi-structured interviews with midwives and traditional birth attendants (TBAs). Eligible participants spoke English, and provided maternity care during COVID-19 in Lagos, Nigeria. Participants were recruited via purposive and snowball sampling, from primary health facilities in seven Local Government Areas of Lagos State. Interview transcripts were analysed thematically following the framework method.

**Results:**

Sixteen midwives (*n* = 11) and TBAs (*n* = 5) were interviewed from March to April 2021. Two overarching themes were identified from the data. ‘Maternity care workers’ willingness and ability to work during the COVID-19 pandemic’ outlined negative influences (fear and uncertainty, risk of infection, burnout, transport difficulties), and positive influences (professional duty, faith, family and employer support). Suggestions to improve ability to work included adequate protective equipment, training, financial support, and workplace flexibility. ‘Perceived impact of COVID-19 on women’s access and uptake of maternity care’ highlighted reduced access and uptake of antenatal and immunisation services by women. Challenges included overstretched health services, movement and cost barriers, and community fear of health facilities. Participants reported delayed healthcare seeking and unattended home births. Midwives and TBAs identified a need for community outreach to raise awareness for women to safely access maternity services. Participants highlighted the responsibility of the government to improve staff welfare, and to implement public health campaigns.

**Conclusions:**

Despite disruption to maternity care access and delivery due to COVID-19, midwives and TBAs in Lagos remained committed to their role in caring for women and babies. Nevertheless, participants highlighted issues of understaffing and mistrust in Lagos’ underfunded maternity care system. Our findings suggest that future resilience during outbreaks depends on equipping maternity care workers with adequate working conditions and training, to rebuild public trust and improve access to maternity care.

**Supplementary Information:**

The online version contains supplementary material available at 10.1186/s12913-022-08009-y.

## Background

Coronavirus disease (COVID-19), caused by severe acute respiratory syndrome coronavirus 2 (SARS-CoV-2) [1], was declared a pandemic on 11th March 2020 by the World Health Organisation (WHO) [2]. The highly transmissible COVID-19 outbreak (global 137 million confirmed cases; 3.09 million deaths by 13th April 2021) challenged health systems to maintain essential healthcare services [3]. Nigeria reported its first case on February 27th 2020. By April 13th 2021, Nigeria had 163,911 infections and 2061 deaths, with Lagos State at the epicentre with 58,014 confirmed cases [4].

A significant concern during the COVID-19 pandemic has been the continuation of the maternal and newborn health services, including antenatal, intrapartum, and postnatal care. Despite being Africa’s largest economy, the Nigerian healthcare system is fragile due to underfunding making it challenging to provide for its population of 200 million [5]. Previous research has indicated that epidemics such as the Ebola virus in West Africa can devastate provision and utilisation of maternity health services in low-resilience health systems, due to resource shortages, mistrust of healthcare systems and fear [6, 7]. Modelling studies [8, 9] have predicted that the indirect effects of COVID-19 on maternal health in Low-to-Middle Income Countries (LMICs) could be severe, with an estimated 8.3–38.6% increase in maternal deaths per month [8]. Such theoretical figures translate into real concern in LMICs including Nigeria, where the pandemic may halt or reverse progress in maternal health [9]. Unprecedented ‘lockdowns’, deepening socioeconomic inequalities [10, 11], and fear of COVID-19 could erode accessibility to healthcare. This highlights the need to support maternity healthcare workers [12, 13].

Maternity care workers are key stakeholders in safeguarding maternal and newborn health, yet they face challenges. Nigeria has a high Maternal Mortality Ratio (MMR) at 512 maternal deaths per 100,000 live births per year [14], accounting for 19% of global maternal deaths [15, 16]. Although the majority of deaths are preventable [17], Nigeria lacks robust maternity care infrastructure, with a shortage of skilled birth attendants, and suffers from low service utilisation, and disparities in access. According to the pre-COVID Nigerian Demographic and Health Survey in 2018, only 57% of Nigerian women had four or more antenatal visits, 43% of women gave birth with a skilled attendant, and 39% delivered in a health facility [14], which some perceive as inaccessible and costly [18]. Many women in Nigeria are cared for by traditional birth attendants (TBAs), defined by the WHO as persons ‘who assist the mother during childbirth and who initially acquired their skills by delivering babies themselves or through apprenticeship to other TBAs’ [19]. Prevalence of TBA-assisted delivery in Nigeria is 20.5% [20]. Although the Nigerian health system does not formally recognise TBAs [21], studies show that women choose to deliver with TBAs as they perceive them as affordable and respectful [22]. Hence, the role of TBAs in women’s health in Nigeria cannot be overlooked. Maternity care guidelines during COVID-19 emphasise continuous care provided by skilled birth attendants, with adaptations to minimise non-urgent contact, including adopting telemedicine to maintain WHO’s standard eight prenatal visits [23, 24]. However, telemedicine is not widely available in Nigeria. COVID-19 threatens to exacerbate barriers of accessing skilled care, thus increasing pressure on health workers.

The existing literature of maternity care workers’ experiences during COVID-19 cites challenges including a physically and emotionally demanding workload, burnout, inadequate personal protective equipment, disrupted service provision and the switch to telemedicine. A global survey of 714 maternal and newborn health professionals highlighted disparities in preparedness, as 82% of maternity providers from High Income Countries received updated guidelines compared to 47% in LMICs [25]. Contemporary studies of COVID-19 in Lagos report maternity providers’ concerns over insufficiently prepared facilities, particularly the availability of personal protective equipment [26]. Existing studies are quantitative, and provide little information on the experiences of maternity health workers in LMICs. Unskilled but frequently-used birth attendants such as TBAs may offer insight into women’s health seeking practices during the COVID-19 pandemic. One quantitative study of TBAs in Lagos during COVID-19 found 37.5% of respondents had inadequate knowledge of infection prevention and control [27]. Therefore, this study aimed to add to the growing evidence base, and explore midwives’ and TBAs’ experiences of COVID-19 in Lagos, Nigeria to gain insight into improving maternity services and pandemic preparation strategies.

## Methods

### Aim

The aim of this study was to explore maternity healthcare workers’ experiences of, and perceptions about providing maternity care during the COVID-19 pandemic in Lagos State, Nigeria. Specific research objectives were to understand the role of midwives and TBAs during the pandemic; barriers and facilitators to delivery of care; and the perceived impact of COVID-19 on maternal and newborn healthcare.

### Design

This generic qualitative study design was driven by the research aims of exploring experiences and perceptions, and as such did not draw on a priori theory, as is consistent with a generic approach [28, 29]. In-depth interviews were used to explore maternity care workers’ experiences of the COVID-19 pandemic. This research study conforms to Consolidated Criteria for Reporting Qualitative Studies (COREQ) guidelines [30].

### Setting

This study was developed in collaboration with a senior public health doctor and researcher with experience conducting qualitative research in Lagos, Nigeria. As the most populous and commercial hub in the country, Lagos State was the epicentre of COVID-19 cases in the country [4]. Due to the local researcher’s prior knowledge and research experience in Lagos State, it was chosen as an appropriate study setting with maternity healthcare workers experiencing the frontline pandemic response. According to UN-Habitat and international development agencies’ estimates, Lagos State had about 24.6 million inhabitants in 2015 [31]. Lagos has a diverse population with many ethnic groups, and commonly spoken languages are Yoruba, Pidgin English, and English [30]. The state comprises 20 Local Government Areas (LGAs), which can be categorised as urban, peri-urban, or rural [5].

### Study participants

Skilled maternity care workers (not TBAs) were purposively selected from seven LGAs (Lagos Mainland, Surulere, Ikeja, Alimosho, Kosofe, Badagry, Oshodi-Isolo), chosen to provide a range of population and geographical contexts [32]. The Medical Officer of Health in each LGA purposively identified maternity care workers in these areas, to provide a diverse participant group according to age, gender, and level of maternity care experience. For TBAs, a convenience sampling approach was taken [33]: study information was shared with all TBAs in the local researcher’s existing professional network with the permission of the Lagos State Traditional Medicine Board [32]. Due to a low response rate, this was supplemented by a snowball sampling approach, where participants were asked to identify colleagues who may be willing to take part, and share study information with them [34]. Eligible participants were over 18 years old, and able to speak English. Potential participants were provided with an information leaflet that outlined the study information, and were invited to interview at their chosen venue, or a venue provided by the local researcher. Reasons for non-participation included being too busy due to involvement in the COVID-19 vaccination programme that commenced in March 2021.

### Data collection

Remote (Zoom software), semi-structured interviews were conducted by the primary researcher based in the UK from March to April 2021. Interviews were in English, audio-recorded and lasted 30–90 minutes. An interview topic guide (see Additional file 1) was developed based on study aims, existing literature and discussion with the research team [35]. The data was collected by [initials anonymised for peer review], a UK-based female medical student and novice qualitative researcher, who is not Nigerian. The work was undertaken as part of a programme of international health study, and pre-COVID this would have involved an academic placement in Lagos and face-to-face fieldwork for a research project. Due to the pandemic, international travel was not possible. To mitigate the lack of local exposure, [initials anonymised for peer review] worked in close contact with [initials anonymised for peer review], a female Nigerian researcher and public health doctor based in Lagos, to develop and pilot the interview questions, and to discuss and reflect on cultural differences and their impact on data collection. The project provided resources (medical student time, research costs funding) to deliver the research, and formal training in qualitative methods was provided. This was complemented by extensive supervision and support of the research process by two academic public health doctors with qualitative expertise (one in Nigeria, one in the UK). Before interview, verbal informed consent was recorded, and the interviewer administered a verbal questionnaire to capture participants’ socio-demographic data and contextualise data. Field notes were written after each interview to reflect on initial impressions and potential themes, data saturation, and to adjust the topic guide.

### Data analysis

An inductive approach was used to generate findings from the data. Thematic analysis was conducted following the framework method outlined by Gale et al [36]*,* to provide a systematic method to identify patterns across and within cases. Firstly, the primary researcher performed verbatim transcription to facilitate familiarisation of data. Three members of the research team independently coded three transcripts, then collaboratively developed a working analytical framework which grouped codes into categories. The primary researcher coded the remaining transcripts, adopting an iterative approach to revise the framework in consultation with other researchers. Data was charted and summarised in a framework matrix in Microsoft Excel, to interrogate data, develop themes, and make within- and cross-case comparisons, and identify deviant cases. Analytical summaries of themes were developed and refined in the context of the research question. Analytical triangulation was used as developing themes were discussed with the research team. Our multicultural, team-based approach to data analysis involved co-authors based in Nigeria and the UK to enhance cultural integrity and maintain researcher reflexivity to examine how researcher’s beliefs, judgements, and practices influenced construction of findings [37]. Data was managed using QSR NVivo 12 [38]. Following data analysis, transcripts were not returned to respondents for comments and corrections.

## Results

16 maternity care workers were interviewed between March and April 2021. The desired number of participants was achieved (midwives, *n* = 11; TBAs, *n* = 5). Table 1 summarises the participant demographics. Participants were aged 27–63 with a mean age of 43.75 years. Participants were from Yoruba and Ogu ethnic groups, and most were Yoruba. Two overarching themes were developed: ‘Maternity care workers’ willingness and ability to work during the COVID-19 pandemic’, and ‘Perceived impact of COVID-19 on women’s’ access and uptake of maternity care’ (See Table 2).

**Table 1:** Participant demographics

Participant demographics	All participants *n* = 16	Frequency
Age range (years)	25–34	2
	35–44	7
	45–54	5
	55–64	2
Gender	Female	14
	Male	2
Occupation	Midwife	3
	Nurse midwife	8
	Traditional birth attendant (TBA)	5
Work experience in role (years)	0–9	4
	10–19	6
	20–29	3
	30–39	2
	40–49	1
Highest level of education	Secondary	2
	Tertiary	14

**Table 2:** Overview of themes, subthemes (with concepts)

Themes	Subthemes
1. Maternity care workers’ willingness and ability to work during the COVID-19 pandemic	Negative influences • Risk of exposure, fear, uncertainty • Stressful working conditions and inadequate support
	Positive influences • Motivation, adaptation, faith • Support, training, preparedness
2. Perceived impact of COVID-19 on women’s access and uptake of maternity care	Challenges to access and uptake of maternity care • Health system weaknesses • Lockdown, poverty, and fear
	Strategies to improve access and uptake of maternity care • Community outreach and collaboration • Adaptation over time • Health system improvements

### Theme 1: maternity care workers’ willingness and ability to work during the COVID-19 pandemic

This theme reflects negative and positive influences on workers’ willingness and ability to work during COVID-19.

#### Negative influences

##### Risk of exposure, fear, uncertainty

All participants identified risk of exposure to COVID-19 as health workers. Subsequent stigma had a negative influence on willingness to work. Participants reported less contact with COVID-19 patients in community care, but recognised the risk of exposure from asymptomatic cases and limited testing.

*“We have [a] high risk of contracting COVID-19 because especially with our work at the community level, we don’t diagnose and we are not treating them”* P7, midwife.

Feelings of fear and uncertainty restricted participants’ ability to work.

*“You have to treat everybody like a positive patient because you don’t know who is who”* P1, midwife.

Some TBAs referred patients for COVID-19 testing before attending to them.

*“We referred many of our patients to the hospital because you don’t know who is who…so you don’t need to damage your life”* P16, TBA.

Participants feared the “killer virus”. Hearing about health worker deaths from COVID-19 negatively impacted willingness to work.

*“Fear of going out to work or going to save lives, you don’t know what can happen…you hear that one of your professional colleagues contracted the virus and the person is physically sick - that gives someone tension”* P5, midwife.

One midwife tested positive for COVID-19. She perceived that her exposure and infection was linked to limited COVID-19 testing for pregnant women before labour, and inadequate personal protective equipment (PPE).

*“You can’t tell a patient that is coming from home to deliver that ‘Madam, go and take COVID test before I can take delivery’. I needed a full PPE which was not provided…I believe I was positive because of the work I am doing…it will be from that labour room”* P11, midwife.

Fear of exposing their own family to coronavirus affected professionals’ willingness to work. Participants frequently adopted disinfection routines when returning home.

*“I don’t even allow them to mingle with me. Entering the house, I remove all my clothes and bathe myself”* P11, midwife.

##### Stressful working conditions and inadequate support

Working during the pandemic was “stressful”. Participants perceived that they were vulnerable to “breakdown” and less able to work effectively.

*“I just want to go and lie down, it’s not easy”* P13, midwife.

Key stressors were high workload, rapidly adapting practices, and inadequate resources. Midwives and TBAs felt less able to work without adequate government support. Some criticised insufficient supplies, a lack of transparency and delayed response. Yet, some participants commented that support improved over time.

*“Planning then is not enough or there may be a delay in transportation…but for now we have everything- then we did not have enough. Our government would have learned their lesson now- they should provide ahead”* P11, midwife.

TBAs felt less supported than midwives and wanted “more training” from the government.

*“We manage ourselves, nobody is supporting us”* P6, TBA.

With inadequate support, many midwives and TBAs bought, re-used, or made their own PPE.

*“Nurses are just using their money to get the face masks, everything they are going to use, so it’s a lot of stress…we are not working with ease, we are not working with peace of mind”* P1, midwife.

Both midwives and TBAs felt less willing to work without financial support. Midwives cited insufficient work “allowance” and TBAs commonly discussed loss of income.

*“No support…they pay just one month allowance…we were just struggling”* P12, midwife.

*“No help from any other place, you have to struggle and starve yourself…you go to home with nothing. They will tell you this work is humanitarian job… are you not going to feed yourself? Are you not going to feed your family?”* P10, TBA.

Finding transport restricted participants’ ability to get to work, and was stressful and costly. Transport for midwives varied between LGAs. Some were transported in “ambulances”, while some commented, *“we are not given that privilege”* P13, midwife.

Some midwives felt less willing to live ‘on-site’ at health facilities during lockdown. One midwife was redeployed to another LGA. Some expressed difficulty raising concerns about working arrangements, feeling that they had no choice than to *“flow with the wave”* P7, midwife.

*“I didn’t expect anybody to transfer me or to post me out of my own community to another place which they did and there’s nothing we can do…I just have to follow instructions”* P1, midwife.

Some participants expressed unwillingness to work with employers unless improvements were made. Participants felt they did not have power to make changes without authority of “people at the top”.

*“I told my boss if you don’t provide me PPE, I’m not going to do any procedure because I can’t expose myself. So, it took us one to two weeks before they gave us some things”* P15, midwife.

#### Positive influences

##### Motivation, adaptation, faith

Participants appeared motivated and willing to work despite challenges. Universally, there was a duty of patient care. To work in circumstances described as “war time”, participants emphasised the need to be resilient and “stand firm”.

*“I accept all those challenges as part of service to humanity”* P2, midwife.

Many participants framed the pandemic as a learning opportunity, apparently increasing their willingness to work. Some took “online courses” to understand COVID-19 with aim to “build a better future” for communities.

*“If I see something I will just Google it and it makes me to be more vigilant…to add to my knowledge”* P15, midwife.

Participants described how their ability to work improved over time. Some cited how professional growth and development enabled them to cope with challenges.

*“COVID-19 has taught me that there is a way to work than the way I was working before, like protecting yourself with PPE and still attending to patients…even in pandemic you can still make impact”* P9, midwife.

Participants frequently referenced faith assisting them to cope with work, expressing trust in the “will of God”.

*“Most important to you is the mother is alive, the baby is alive; every other thing…God will put it in place”* P1, midwife.

One TBA believed the pandemic was a test to be “holy” and pray.

##### Support, training, preparedness

Participants derived support from family, friends, and social media.

*“Parents were there to support me…nothing like stigmatisation…that alone gives me confidence that I can work and survive through the pandemic”* P5, midwife.

Practical and moral support from employers and government increased willingness and ability to work, including provision of PPE, food supplies for workers, and transportation.

*“Partners with [the] primary healthcare board make provision for all these precautionary gadgets for us… glove, nose mask and the rest”* P2, midwife.

Non-governmental organisations (NGOs) were perceived as being helpful to midwives and TBAs in providing resources and training, but less-so early in the pandemic.

*“NGOs they really try during that time…sanitiser, gloves, goggles they give us, and it’s really helped.”* P1, midwife.

Midwives and TBAs received training from local governments on COVID-19 and infection prevention control. This was seen to “widen knowledge” and improve their preparedness and ability to identify COVID-19 cases and adapt facilities.

*“They train us. That is why we are able to train our patients to teach them how to wash [their] hands…but we want more training”* P14, TBA.

Some TBAs described mobilisation by the government to deliver community surveillance, for which they were paid.

*“The government sees us to be visiting houses during the pandemic…they compensate us by giving us some change [money] to support ourself”* P16, TBA.

Midwives suggested that the government should improve staff welfare, including financial support, to improve willingness and ability to work. Participants rationalised that if staff worked in a more “conducive environment” e.g., functioning electricity and lighting, this could improve their ability to “tend well” to patients.

*“If the government will rise to the need of that person, you know you want to serve your nation with all your heart, you want to serve the people with everything you have”* P1, midwife.

### Theme 2: perceived impact of COVID-19 on women’s access and uptake of maternity care

This theme reflects provider’s perceptions regarding challenges and strategies to improve women’s access and uptake to maternity care during COVID-19.

#### Challenges of access and uptake to maternity care

##### Health system weaknesses

Midwives perceived that access to skilled maternity care was difficult, because antenatal appointments for non-urgent cases had been reduced, and face-to-face home visits were not possible.

*“We have to use our home visit as a phone call. When you see somebody physically it’s different from hearing the voice”* P8.

Some midwives perceived that the pandemic had diverted the focus away from maternity care. Nurse midwives described taking on additional roles, such as administering COVID-19 vaccines.

*“It really disturbed maternal health care because the same space we’re using for our clinic has been acquired by COVID-19 vaccination”* P9.

In primary care, midwives described initial confusion about appropriate modifications to practice.

*“When the thing first came, we thought it’s not going to last long, so even immunisation for babies we were skipping some because we don’t want crowd”* P9.

Participants described additional, pre-existing access problems in Nigeria’s health system, such as understaffing and weak communications infrastructure.

*“In Nigeria it’s not easy for us to do telemedicine…because it’s not even everybody that can afford an Android phone”* P5, midwife.

##### Lockdown, poverty, and fear

Midwives and TBAs frequently reported that patient uptake of their services had reduced, with a decline in institutional births, antenatal registrations, and maternal and neonatal immunisations.

*“It was so bad, so the targets both on immunisation, antenatal, family planning and everything dropped. The target is just picking up again”* P13, midwife.

Participants explained that lockdown movement restrictions, financial inaccessibility, and changed family spending priorities had reduced women’s access to care.

*“No job during this period, it’s lockdown…people are staying at home…so to spend money in the hospital they find it difficult, it’s only those that have complications that come”* P12, midwife.

Stigma, and community fear of COVID-19 and health facilities were considered major challenges to healthcare seeking. Midwives and TBAs stated that patients feared they would “catch COVID at the hospital” or be incorrectly “tagged” as positive and taken to isolation centres.

*“They don’t want to come to the clinic because they are afraid of contracting the virus and spreading it to their loved one at home”* P7, midwife.

*“They believe that you will call on the COVID-19 expertise [experts] to come and take them away to quarantine them”* P10, TBA.

Misinformation that COVID-19 was a “scam” or ploy to “embezzle money” was perceived to fuel distrust in government facilities. TBAs reported that some clients did not believe in COVID-19:

*“Most of them they did not believe, they are telling me it’s a lie”* P4, TBA.

Midwives perceived that there had been limited uptake of skilled maternity services due to patient preference for TBAs or “faith-based homes”. TBAs suggested this was because they “bear costs” for patients who can’t afford care.

*“It’s a wrong notion for [patients] to think that even at TBA’s place that you cannot contract coronavirus, and that is why they will prefer them [TBAs] than coming to us”* P9, midwife.

Some midwives were critical of TBAs, believing that they exceeded their limits and caused harm.

*“[TBAs] go beyond their limits, they are not doing the proper things…they even infect some people with some other diseases during the delivery process and they even cause high rate of maternal and child death…I only discourage people even in my area not to go there because some of them are trained but some are not well trained”* P13, midwife.

Due to challenges to access, both midwives and TBAs suggested that women delayed health seeking and stayed home. One TBA reported that some pregnant women delivered unattended at home *“without thinking about the risk”* P10. A midwife recalled a patient who *“lost her baby”.* P5.

*“We opened after the lockdown for immunisation, some even come with their baby to show that, ‘I’ve delivered at home, check my baby’…their mother did that, their sister-in-law helped them to ligate the cord”* P11, midwife.

Some providers believed that reduced contact and fear during the pandemic affected their relationship with patients, creating another challenge to maintaining access.

*“There is not much love…not like the relationship before and it’s affecting both patients and nurses’* P3, midwife.

*“People will fight me. They will talk to me in the way I do not even like, but as a community birth attendant I have to be patient”* P4, TBA.

Nonetheless, a few midwives deviated from the consensus, and perceived that access to primary health centres had continued during the pandemic due to closure of secondary facilities.

*“My primary healthcare [centre]… COVID-19 doesn’t affect us because people came from different angles to register, because of the issue that in lockdown that the general hospital did not close down, and the teaching hospital, some of them closed”* P15, midwife.

Many providers perceived that access to healthcare fluctuated with COVID-19 restrictions, and had improved since the easing of lockdown.

*“We have to relax on all those rules now…at that time the number of deliveries was a bit low…but now I think we are balanced, we have bounced back”* P9, midwife.

#### Strategies to improve access and uptake of maternity care

##### Community outreach and collaboration

Midwives suggested that access to skilled maternity care could be improved through community outreach. This aimed to raise awareness about timely access to care, address misconceptions surrounding COVID-19, and educate about prevention. Participants identified that outreach could be facilitated by clear communication through various media, by patients sharing experiences, and through work to build community trust.

*“Media, radio, television more awareness, jingle, health education that a pregnant woman needs to deliver at hospital…clarity will come to the clients, their fear will be allayed…so community awareness of disadvantage of delivering at home…the danger in it”* P11, midwife.

Midwives felt responsible for developing trust with patients, using empathy and communication to address barriers to access.

*“You make them realise in the language that they can understand that though they might see face masks as inconveniencing or whatever, they have to do it”* P2, midwife.

TBAs also assisted access to skilled maternity services, and reported rumours from their community about COVID-19 to policymakers.

*“I am like a gatekeeper between the government and the community, so any information coming related to health that is brought to the committee – I just engage it to them”* P4, TBA.

Initiatives from government and NGOs were perceived to be helpful to motivate women to access skilled maternity care. Lagos government provided free antenatal care to women during lockdown.

*“They give them free of charge for those that came for antenatal; they gave them prenatal care drugs”* P15, midwife.

Another midwife used maternity kits donated by NGOs before the pandemic.

*“We have these maternal kits so we give support to pregnant women…it’s another way to motivate them to come…we call them Mama kits”* P13, midwife.

Collaboration between midwives and TBAs was recognised to improve access to skilled maternity care and “reduce maternal and infant mortality”*.* Midwives recognised the influence of TBAs in society, hence primary health centres contacted TBAs and strengthened referral pathways with assistance of NGOs. TBAs perceived a “cordial relationship” with midwives and appreciated their support.

*“We cannot say that [TBAs] should go away; they are not relevant because [patients] will go and meet them and because they are closer to them - more than us, some of them are in their streets, so that’s the reason why health educator call those TBAs and advise them”* P15, midwife.

*“I am one of the mentees mentoring the TBAs; you see we are working together…we build rapport…to arrange for patient to come and meet us at our various clinics”* P2, midwife.

##### Adaptation over time

Midwives and TBAs recognised that patients found it difficult to adjust behaviours, especially infection prevention and control. However, participants discussed that repeated reminders, such as “no mask, no entry”, helped patients learn to adapt and access care safely.

*“Change is always difficult, telling them to do this do that…by the time we reinforce it tell them over and over again they will be used to the system like us”* P9, midwife.

##### Health system improvements

Midwives suggested increasing staffing in government health facilities to improve patient access to skilled providers. TBAs agreed, as they referred to skilled providers for assistance.

*“Government can please help them in the health centre, let them get enough staff so that they can help us traditional birth attendants at centre. We call them and they will come to our aid…because we rely on them”* P16, TBA.

Some midwives discussed the benefits of telemedicine to give patients remote access to care and information. A few participants described using group “WhatsApp” chats to offer patients peer support.

*“COVID brought out the issue of this WhatsApp platform where we communicate with them, tell them to come that the hospital is safe, we are there for them”* P13, midwife.

## Discussion

This study found that COVID-19 created negative and positive experiences for midwives and TBAs in Lagos State, and identified areas to improve support for maternity care workers. Participants perceived that COVID-19 exacerbated challenges to women’s access and uptake of maternity care, and suggested methods to improve access to skilled maternity care. These findings support and add depth to global literature of this emerging topic.

### Suggestions to improve support for maternity care workers

Following United Nations Population Fund’s (UNFPA) stance to ‘maintain a healthy workforce’ [12], our study highlights how maternity care workers in Lagos could be better supported during pandemics. Midwives and TBAs sampled in our study identified key challenges to their willingness and ability to work during COVID-19, including fears about personal risk of infection, uncertainty, inadequate PPE, lack of support, stress, and burnout. These findings are consistent with a global survey [25], and also align with a 2020 survey of maternity providers in Lagos which found 87.2% had experienced burnout since COVID-19 [26]. Evidence shows that providers have been vulnerable to significant mental stress during COVID-19 [39, 40]. Psychological interventions could be used to reduce anxiety, cultivate resilience, and support mental wellbeing of health workers during highly stressful events such as pandemics [41]. A systematic review highlighted that further research is needed to determine the effectiveness of interventions to support the resilience and mental health of frontline workers during pandemics [42]. Most participants in our study were female, and they described specific challenges of fear of infecting family and child-caring responsibilities. This finding is in accordance with literature that suggests that female health workers have disproportionate family responsibilities, which could exacerbate physical and emotional burdens during COVID-19 [43, 44].

Consistent with our study, research into public health emergencies has identified that maternity care workers’ sense of duty overrides challenges, and is a motivator for willingness to work [45]. Our study suggested that willingness to work was strengthened by commitment to patient care, faith, and family support. Given that data collection occurred one year after the pandemic started, a positive finding among participants was professional development, including the ability to “learn a new way of living” to cope with challenges. This temporal change is reflected in literature that suggests that processes of adaptation and personal development can build resilience and ability to cope with stressful conditions [46, 47]. Individual-led coping strategies should be accompanied by organisation-led interventions to improve willingness and ability to work. A recent qualitative study of Indonesian midwives found that adequate protection through PPE availability, effective training and wellbeing support is needed to support midwives in providing maternity care during the pandemic [48]. Maternity providers suggested that Lagos State Government could increase preparedness by providing adequate PPE and training, financial support, transportation for workers, and COVID-19 testing for health workers and pregnant women. Similar to a global survey [25], maternity providers in Lagos reported difficulties in reaching their workplace due to travel restrictions. A key enabler for ability to work was government provision of transport for maternity workers [49]. Participants referred to hierarchies of power within the health system, particularly a lack of decision-making abilities for nurses and midwives, which limited their choices in working conditions and redeployment [50]. Health leaders should create a “conducive environment” to raise concerns and allow workplace flexibility. UNFPA has highlighted the need to expand the global maternity care workforce and invest in midwife-led improvements to maternity service delivery, particularly midwifery leadership and governance [51]. Maternity care workers are influential advocates at community-level, and should be given adequate representation in leadership roles to guide health policies [52]. Given significant ‘brain drain’ of the Nigerian health workforce, the government should focus on improving working environments and satisfaction to increase retention and capacity to cope with future outbreaks [53, 54].

### Maintaining access to maternity care during COVID-19

Despite WHO guidelines to maintain access to essential maternity services during the pandemic [55], a combination of physical, financial, healthcare and social factors limited access and uptake of maternity care in Lagos [49]. Participants in our study described key barriers, including movement restrictions, transportation, and financial inaccessibility. This was corroborated by a survey of women aged 15–49 years in Lagos, with other challenges being fear of contracting COVID-19, and mandatory face mask use at facilities [56]. Our study aligns with global concerns about the indirect effects of the pandemic on increased risk of maternal and newborn complications and mortality, particularly in low resource settings [8]. Maternity workers in our study described delayed health seeking, missed immunisations, and reduced antenatal registrations. Similar findings were observed in other LMICs during COVID-19, such as reduced institutional births and increased stillbirth rate in Nepal [57], and community fear of facilities in India [58]. Maternity systems must be strengthened to avoid persistent reduced uptake of skilled maternity services as was seen following Ebola [6]. A global survey reported the negative impact of COVID-19 on respectful maternity care e.g. compromised standards of care, staff overwhelmed, reduced emotional and physical support for women [59]. This warrants further exploration of current and long-term effects of COVID-19 pandemic on maternity systems and health seeking behaviours, both in Nigeria and other LMICs.

Participants suggested that the Lagos Government’s strategies to control COVID-19 should integrate approaches to improve access to maternity services. Maternity workers reported that financial difficulties were widespread during COVID-19, as evidenced by telephone surveys of Nigerian households that found opposition to strict lockdowns, because they exacerbate socioeconomic hardship [60]. The Lagos State Government addressed financial barriers and provided free antenatal, delivery and laboratory services at primary healthcare centres during lockdown [56]. Pre-COVID, LMICs including Nigeria relied on external support from NGOs e.g., maternity kit donations, to incentivise access to skilled maternity care. The role of NGOs during the COVID-19 pandemic could be further researched. Midwife participants commented that health worker shortages and new COVID-19 vaccination duties reduced their ability to run routine maternity services. To remedy this, governments should increase staff recruitment, and avoid redeployment of maternity providers to non-maternity care roles [61].

Based on our study findings and consistent with global literature [58], strategies to “allay fears” about COVID-19 and build trust in health systems during a public health crisis are key to maintaining access to skilled maternity care in Lagos. Participants recommended community outreach via various media (television, radio, social media) to address fears and stigma around COVID-19, encourage attendance of government facilities and strengthen patient-provider relationships. Midwives reported instances of women having unsupervised home deliveries, or preference for ‘unskilled’ TBAs. Patient preference for TBAs could be influenced by embedded traditions, as a survey of Nigerian households found 40% of respondents believed that COVID-19 could be cured by herbal remedies [60]. Considering TBAs’ influential role in local communities, government and NGOs could engage TBAs as health promoters during public health outbreaks, and bridge the gap between community and skilled maternity providers [62]. Community participation is essential in designing community health programmes during public health outbreaks such as COVID-19, which was highlighted by a study into the willingness and barriers to receive the COVID-19 vaccine among residents in the UK and Nigeria [63]. Adding to a quantitative study of TBAs in Lagos [27], our qualitative interviews with TBAs reflected a desire for more government training to develop knowledge of COVID-19 infection prevention and control. Referral pathways to skilled maternity services could be strengthened through mentoring of TBAs, and increased staffing [64]. Collaboration between skilled maternity providers and TBAs could raise awareness about timely access to skilled care, to reduce risk to mother and baby, and improve public trust and satisfaction in maternity services [56].

COVID-19 challenges the status quo, as telemedicine could provide remote access to care and peer support through digital communication platforms such as WhatsApp. However, COVID-19 heightens global disparities [54], with limited telemedicine capacity in Lagos due to infrastructural constraints and cost barriers [65]. A qualitative study among the general Nigerian population during the COVID-19 pandemic revealed the importance of remote access to care, and potential barriers to telemedicine including “poor internet service”, “concerns about confidentiality”, and “technological illiteracy” [66]. Lagos State has launched the ‘Eko Telemed’ service as part of the new Health Scheme; however, this would need to be scaled up to fill this gap [67].

Despite perceptions that maternity services were “picking up again”, subsequent highly contagious COVID-19 Delta and Omicron variants have propelled surges of COVID-19 infections in Nigeria since July 2021. Global COVID-19 vaccine distribution inequity means that Nigeria’s vaccination rate falls behind wealthier countries, with four million vaccine doses received between March and August 2021 [68]. In addition to COVID-19, underutilisation of skilled attendants, poverty, and epidemics remain key obstacles to progress in maternal health in Nigeria [69]. Aligning with WHO guidance, this study recommends that policymakers protect gains in maternal health, and prepare health systems and staff for future epidemics [70]. Maternal indicators are sensitive markers of health system resilience, and close monitoring of provision and utilisation of services in terms of equity, access, coverage, and quality is required [51].

### Strengths and limitations

Strengths: We have not identified previous qualitative studies of maternity care workers in Nigeria in the peer-reviewed literature. Inclusion of midwives and TBAs gives broader insight into the impact of COVID-19 on maternity healthcare in a context where many women use the services of TBAs, although it is recognised that TBAs are not skilled birth attendants. Data saturation was achieved, with similar concepts repeated in the data.

Limitations: TBAs are highly heterogeneous; however, it was only possible to recruit TBAs registered with Lagos State Traditional Medicine Board. Unregistered TBAs are likely to be less connected to the health system and may offer different perspectives of the pandemic. Resource constraints meant that those who could not speak English were excluded, which may have reduced the diversity of participants. This work was only possible due to funding of CL as part of an international health study programme, and local research staff capacity was not available to undertake data collection. Cultural differences between the primary researcher (female, British, medical student) and participants may have impacted on data collection and interpretation of data. While impossible to eliminate completely, to mitigate its impact the research team worked closely throughout, reviewed audio and transcribed data, met frequently, and adopted a reflexive approach, drawing extensively on input from TO, an academic Lagos-based public health doctor with experience of both the local context, and qualitative research. CL was a novice qualitative researcher, but received formal training and close supervision by TO and BT (an experienced qualitative researcher) throughout. Remote data collection was generally successful but technical issues compromised some audio quality and non-verbal cues. Lagos State has comparatively generous resources for health, reducing transferability of findings to other regions of Nigeria [71].

## Conclusions

Midwives and TBAs offered contemporary insight into improving maternity care in Lagos during COVID-19. Employers and policymakers should involve maternity care workers in decision-making to address training, PPE provision, and financial needs. Organisation-led support is urgently needed to improve worker retention amidst staff shortages. COVID-19 has highlighted the fragility of maternal healthcare access in Nigeria. Community outreach to address public fear, health advocacy from maternity care workers, and telemedicine were recommended to improve women’s access and uptake of maternity care during outbreaks. Although this study found that maternity care workers adapted to new challenges during COVID-19, subsequently Nigeria faced new waves of COVID-19 infections and uncertainty. Slow vaccine rollout, economic disparities, overstretched healthcare systems, and public mistrust of government hinder maternal health progress. Further studies are needed to evaluate the long-term effects of COVID-19 on maternity healthcare systems, and on women’s health seeking behaviours. This study can inform policymakers’ efforts to strengthen maternity care systems, protect providers, and sustain access to care during current and future pandemics. This is important to maintain progress in maternal and newborn health in Nigeria and globally.

## Supplementary Information

**Additional file 1.** Interview Topic Guide. Interview topic guide questions used for semi-structured interviews.

## Data Availability

The authors did not seek ethical permission from the participants, nor the ethics committee, for the data to be used for anything other than this particular research study. The authors do not have explicit permission for data sharing, re-analysis nor future studies and so would be inappropriate and unethical to make them available in the public domain. While anonymised, the data contains potentially identifying patient information. However, qualified individuals can direct queries by contacting Dr. Ruth Riley (r.riley@bham.ac.uk) – chair of the University of Birmingham BMedSci Intercalation Internal Ethics Review Committee.

## Abbreviations

COVID-19: Coronavirus disease 2019
WHO: World Health Organisation
LMIC: Low-Middle-Income Country
HIC: High-Income Country
TBA: Traditional Birth Attendant
PPE: personal protective equipment
NGO: Non-Governmental Organisation
UNFPA: United Nations Population Fund
