# Supplementary material for: Perceptions and experiences of maternity care workers during COVID-19 pandemic in Lagos State, Nigeria; a qualitative study
Source: BMC Health Serv Res. 2022 May 6;22:606. doi: 10.1186/s12913-022-08009-y (PMC9073449; doi:10.1186/s12913-022-08009-y)
Supplement: Supplementary file 1 — Additional file 1. Interview Topic Guide. Interview topic guide questions used for semi-structured interviews. [file 12913_2022_8009_MOESM1_ESM.docx]

Additional file 1: Interview Topic Guide

**“First, I want to ask you about your work and the care you provide. I understand you are a midwife/TBA, please can you tell me about your role?”**

1. **Background of maternity care provider: Please can you tell me about your role?**
   - Probe: What type of maternity care do you provide?
   - Probe: Who do you provide care for?
   - Probe: How did you feel about your role before the pandemic?
2. **Perceptions of COVID-19: What do you know about the COVID-19 pandemic?**
   - Probe: How did you feel about COVID-19 when cases started to rise in Lagos?
   - Probe: How do you currently feel about the COVID-19 pandemic in Lagos?
3. **Experiences of COVID-19: Can you describe your experience working during the COVID-19 pandemic?**
   - Probe: Have you ever experienced anything like this before?
   - Probe: Have you been in contact with patients with suspected/confirmed COVID-19?
   - Probe: How do you feel caring for them?
4. **How has COVID-19 outbreak affected maternity care services?**

- What impact do you feel that COVID-19 pandemic has had on your work?
  - Probe: How has COVID-19 affected normal daily tasks/responsibilities?
  - Probe: How has COVID-19 affected the way you look after patients?
  - Probe: Have supply of drugs, equipment, PPE been affected?
  - Probe: Have staff been redeployed from or within your facility?
- Please tell me about any changes COVID-19 has had on your work
  - Probe: Can you explain why there have been these changes?
  - Probe: How do you feel about the changes you have had to make during the pandemic?
  - Probe: COVID-19 control measures - How do you feel about mask wearing/social distancing/handwashing?
- What differences have you noticed in the way that patients interact with your services during COVID-19?
  - Probe: Has there been a change? Why?

1. **What preparations were put in place where you worked?**
   - Probe: Did you feel prepared?
   - Probe: What do you feel was particularly successful?
   - Probe: Can you describe training?
2. **Perceived challenges and barriers: What challenges have you experienced in providing usual maternity care to patients?**
   - Probe: What has been the hardest part of working during the pandemic?

- Is there anything that helps you cope with these challenges?
- If you have concerns who do you raise them with?

1. **Perceived facilitators and improvements: What could be done to better support you during the pandemic?**

- How supported did you feel while working during the pandemic?
  - Probe: From local health system? Government? TBA/Midwifery association?
- What has helped you to continue working during the pandemic?

1. **Reflection on improvements: how could maternity care services be strengthened during the outbreak?**

- What could be done to improve looking after women/babies during the pandemic?
- How do you feel about formal/informal (midwives/TBAs) services?
- Is there anything you feel should be changed to make health services more effective in future emergencies?
- Who is responsible for making these improvements?

1. **Personal reflection: On reflection, what impact do you feel COVID-19 had on you?**
   - Probe: How are you managing?
   - Probe: What do you think about the consequences of working in this pandemic?
   - Probe: What have you learned?
2. **Final points: Is there anything else you would like to mention that you feel is important?**

***Thank you very much for your time and sharing your experiences and opinions with us***
